# 2‐Arachidonoylglycerol‐mediated endocannabinoid signaling modulates mechanical hypersensitivity associated with alcohol withdrawal in mice

**DOI:** 10.1111/acer.14949

**Published:** 2022-10-12

**Authors:** Amanda Morgan, Danielle Adank, Keenan Johnson, Emily Butler, Sachin Patel

**Affiliations:** ^1^ Department of Psychiatry and Behavioral Sciences Northwestern University Feinberg School of Medicine Chicago Illinois USA; ^2^ Vanderbilt Brain Institute, Vanderbilt University Nashville Tennessee USA; ^3^ Interdisciplinary Program in Neuroscience Vanderbilt University Nashville Tennessee USA

**Keywords:** addiction, analgesia, cannabinoid, CB_1_, CB_2_, pain

## Abstract

**Background:**

Alcohol use disorder (AUD) commonly occurs in patients with chronic pain, and a major barrier to achieving abstinence and preventing relapse is the emergence of hyperalgesia during alcohol withdrawal. Elucidating novel therapeutic approaches to target hyperalgesia associated with alcohol withdrawal could have important implications for treating AUD. Here, we examined the role of 2‐arachidonoylglycerol (2‐AG)‐mediated endocannabinoid (eCB) signaling in the regulation of hyperalgesia associated with alcohol withdrawal in mice. We tested the hypothesis that pharmacological augmentation of 2‐AG signaling could reduce hyperalgesia during withdrawal.

**Methods:**

Male and female C57BL/6J mice were tested during withdrawal from a continuous access two‐bottle choice (2BC) paradigm to investigate how eCB signaling modulates mechanical and thermal sensitivity during withdrawal. Mice were pretreated with the monoacylglycerol lipase (MAGL) inhibitor JZL184 to elevate levels of 2‐AG. Rimonabant or AM630 were given to block CB_1_ and CB_2_ receptor activity, respectively. DO34 was given to reduce 2‐AG by inhibiting the 2‐AG synthetic enzyme diacylglycerol lipase (DAGL).

**Results:**

After 72 h of withdrawal, male and female mice exhibited increased mechanical, but not thermal, hypersensitivity, which normalized by 7 days. This effect was reversed by pretreatment with JZL184. The effects of JZL184 were prevented by coadministration of either the CB_1_ or the CB_2_ antagonist. DO34, Rimonabant, and AM630 exacerbated mechanical hypersensitivity during alcohol withdrawal, causing an earlier onset and persistent hypersensitivity even 1 week into withdrawal.

**Conclusions:**

Our findings demonstrate the critical role of 2‐AG signaling in the bidirectional regulation of mechanical sensitivity during alcohol withdrawal, with enhancement of 2‐AG levels reducing sensitivity, and inhibition of 2‐AG signaling exacerbating sensitivity. These data suggest that 2‐AG augmentation represents a novel approach to the treatment of alcohol withdrawal‐associated hyperalgesia and AUD in patients with comorbid pain disorders.

## Graphical abstract

The endocannabinoid 2‐Arachidonoylglycerol bidirectionally regulates mechanical hypersensitivity associated with alcohol withdrawal in mice. Systemic pharmacological enhancement of 2‐AG levels with the MAGL inhibitor JZL184 reduces mechanical hypersensitivity during alcohol withdrawal, and the effect of MAGL inhibition is prevented when blocking both CB1 and CB2 receptors. Furthermore, preventing synthesis of 2‐AG with a systemic pharmacological inhibitor of DAGL exacerbates sensitivity during alcohol withdrawal. These data suggest 2‐AG augmentation could represent a novel approach to the treatment of alcohol withdrawal‐associated hyperalgesia and AUD in patients with comorbid pain disorders.

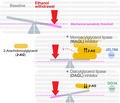

## INTRODUCTION

The lifetime prevalence of alcohol use disorders (AUDs) is ~30% in the U.S. population (Grant et al., 2015), with an estimated 15.5 million U.S. adults suffering from AUDs (SAaMHS Administration, 2021). Disorders of acute and chronic pain often co‐occur with AUDs, with chronic pain affecting an estimated 114 million adult Americans (Egli et al., 2012, Grant et al., 2004). Alcohol has analgesic properties, with 38% of heavy drinkers reportedly drinking to treat pain (Alford et al., 2016). Drinking to cope with physical pain can be effective initially due to the analgesic properties of alcohol (Thompson et al., 2017). However, the analgesic effects of alcohol are brief and dose‐dependent, with tolerance emerging relatively quickly (Maleki et al., 2019; Thompson et al., 2017). These pharmacological properties of alcohol can lead to physiological dependence on alcohol for pain relief, contributing to negative reinforcement driven alcohol use (Apkarian et al., 2013; Egli et al., 2012). Importantly, neurotoxic effects of alcohol can result in neuropathy after chronic use. Furthermore, alcohol withdrawal is associated with the emergence of hyperalgesia (Jochum et al., 2010; You et al., 2020) and allodynia which can persist after weeks or months of abstinence (Jochum et al., 2010). Moreover, this effect is seen in patients who are alcohol dependent but report no preexisting chronic or acute pain conditions. For example, pain‐free patients may develop a new heightened sensitivity to painful stimuli when they undergo withdrawal from alcohol after prolonged use (Egli et al., 2012, Jochum et al., 2010). This effect has been well documented in preclinical studies where alcohol withdrawal‐induced hyperalgesia is extensively reported in rodents (Alongkronrusmee et al., 2016; Dina et al., 2006; Fu et al., 2021; Gatch, 1999; Gilpin et al., 2021; Smith et al., 2016).

Major barriers to achieve abstinence and preventing relapse include the emergence of negative affect and increased pain sensitivity during alcohol withdrawal (Egli et al., 2012). Effective pharmacological treatments targeting the interdependence between alcohol and pain are currently limited. For example, Gabapentin is used to treat both acute (Dauri et al., 2009; Ho et al., 2006) and chronic pain conditions (Wiffen et al., 2017) and has recently emerged as a possible pharmacotherapeutic for AUDs (Andrade, 2020). The Federal Drug Administration has approved the drugs disulfiram, acamprosate, and naltrexone to treat AUDs; however, clinical data indicate these medications are only partially effective and have high rates of relapse upon discontinuation of the drugs (Swift & Aston, 2015). None of the medications approved to treat AUDs are useful for the treatment of pain associated with alcohol withdrawal. These data underscore the need to identify new potential therapeutic approaches for the treatment of alcohol withdrawal‐associated hyperalgesia, which could facilitate abstinence by reducing negative reinforcement driven alcohol use in treatment‐seeking individuals.

One emerging target for the treatment of pain is the endogenous cannabinoid (eCB) system, which acts throughout the neural nociceptive system to produce antinociceptive and analgesic effects (Guindon & Hohmann, 2009; Woodhams et al., 2017). The eCBs 2‐Arachidonoylglycerol (2‐AG) and N‐arachidonoylethanolamine (AEA) exert antinociceptive effects in rodents through activation of CB_1_ and CB_2_ receptors (Sagar et al., 2009). These eCBs are also implicated in setting nociceptive thresholds and regulating tonic inhibition of pain responses (Sagar et al., 2009). Pharmacological inhibition of the major 2‐AG catabolic enzyme monoacylglycerol lipase (MAGL) results in increased brain and peripheral levels of 2‐AG and exerts analgesic and anti‐allodynic effects of in a variety of preclinical models via CB_1_ and CB_2_ receptor (Ghosh et al., 2013; Guindon et al., 2011; Hohmann, 2007).

Several techniques for modeling aspects of AUDs in rodents are commonly used, each with some limitations. For example, chronic intermittent exposure in vaporized EtOH chambers, injected EtOH, and the drinking in the dark (DID) are all involuntary consumption models (Holleran & Winder, 2017). The two‐bottle choice method was used here in part for translational value because it allows voluntary access. While withdrawal effects during forced abstinence from EtOH are well‐established in the 2BC model, they are typically not severe or life‐threatening (Holleran & Winder, 2017). The withdrawal effects seen in the 2BC model include depression‐like behavior measured with the forced swim test and novelty‐suppressed feeding test (Holleran et al., 2016; Holleran & Winder, 2017). Importantly, mechanical hyperalgesia has also been reported in mice at 72 h of withdrawal from EtOH using the 2BC administration model (Quadir et al., 2020, 2021), and thus, this model was chosen for this study. Here, we test the hypothesis that 2‐AG signaling regulates pain sensitivity during alcohol withdrawal. We report here that pharmacological augmentation of 2‐AG during alcohol withdrawal reverses mechanical hypersensitivity associated with alcohol withdrawal in mice via actions at both CB_1_ and CB_2_ receptors. Conversely, pharmacological 2‐AG depletion worsens and extends the time course of hyperalgesia observed during alcohol withdrawal, suggesting a critical role for endogenous 2‐AG to counteract hyperalgesia during withdrawal. These data provide new insight into the mechanisms underlying alcohol withdrawal‐associated hyperalgesia and suggest 2‐AG augmentation could represent a novel treatment approach of AUD in patients with comorbid pain disorders.

## MATERIALS AND METHODS

### Animals

C57BL/6J (Jackson Laboratory, ME) female and male mice were between 6 and 7 weeks old at the beginning of experiments. The animal care facilities at Vanderbilt University (Nashville, TN) housed mice in climate‐controlled colony rooms, maintained at 21 ± 2°C, 30% ± 10% relative humidity on a 12L:12D cycle, and with lights on at 0600 h. Food and water were provided ad libitum (LabDiet 5001; LabDiet) for the duration of the experiments. All behavior experiments were conducted during the light phase. All studies were carried out in accordance with the National Institute of Health Guide for the Care and Use of Laboratory Animals and approved by the Vanderbilt University Institutional Animal Care and Use Committee (#M1600213‐01).

### Drugs

The MAGL inhibitor JZL184 (10 mg kg^−1^, Cayman Chemical, MI, USA), CB_1_R inverse agonist Rimonabant (3 mg kg^−1^ APIChem, Hangzhou, Zhejiang, China), and CB_2_R inverse agonist AM630 (3 mg kg^−1^, Cayman Chemical, MI, USA) were prepared in DMSO and injected i.p.at 1 μl g^−1^ bodyweight. DAGL inhibitor DO34 (50 mg kg^−1^, Glixx Laboratories Inc., MA) was administered in a vehicle mixture of EtOH (Pharmco, KY, USA): kolliphor (Sigma‐Aldrich, WI,): saline (Hospira, IL, USA) [1:1:18] was injected at a volume of 10 μl g^−1^ bodyweight. All drugs were administered 2 h before behavior testing, as previous work has shown 2 h after injection, JZL184 10 mg kg^−1^ causes peak increases in 2‐AG (Long et al., 2009) and DO34 decreases 2‐AG (Bedse et al., 2017; Ogasawara et al., 2016). We have also shown JZL184 10 mg kg^−1^ produces behavioral effects after 120 min (Bedse et al., 2017), as does DO34 50 mg kg^−1^(Bluett et al., 2017; Cavener et al., 2018). At doses of 3 mg kg^−1^, both Rimonabant and AM630 have been shown to inhibit cannabinoid‐driven antinociception (Guindon et al., 2013; Lichtman & Martin, 1997). When CB_1_ and CB_2_ antagonists were paired with JZL184, drugs were co‐administered 2 h before testing. In subsequent experiments with Rimonabant and AM630 alone, drugs were administered 2 h prior to testing. 190 proof ACS/USP grade grain‐derived EtOH (Pharmco, KY, USA) was used to for EtOH drinking solutions.

### Two‐bottle choice EtOH drinking and withdrawal model

Mice were first acclimated to single‐housed two‐bottle choice (2BC) cages for 7 days, with two sippers and access to tap water only. For EtOH mice, one bottle of tap water was replaced with 3% EtOH in tap water for 4 days. On the 5th day, the EtOH concentration increased to 7% for 7 days. On the 12th day, the EtOH bottle concentration increased to 10% or 20% and was continuous at this concentration until testing or forced abstinence. EtOH bottle placement (left or right sipper) was rotated weekly. For withdrawal tests, after 21 days of drinking in 2BC cages with either one bottle of 10% or 20% EtOH, the EtOH bottle was replaced with tap water. Control mice were housed in the same conditions for the duration of the experiment with two bottles of tap water.

Mice undergoing EtOH withdrawal were kept physically separated from control mice in the facility to prevent social transfer of pain sensitivity, by use of adjoining enclosed housing rooms (Smith et al., 2016).

### Pain assays

Mechanical punctate sensitivity was assessed by manual application of Von Frey filaments of varying forces (0.4–4.0 g) using the “ascending stimulus” method (Deuis et al., 2017). Mice were tested in groups of four in a plexiglass testing rack, which had four clear plexiglass experimental cubbies boxes. Because mice were single housed for a protracted period, the interior walls of each cubby were covered with a thin piece of dark plastic, so the mice could not see each other during habituation and testing. Each mouse had left hind paw tested first, with increasing grams force monofilaments applied until a positive response was observed. “Positive response” included of paw withdrawal, paw licking, or shaking, either during application or immediately after the filament was removed. The grams force of the von Frey filament that elicited a positive response was designated as the mechanical withdrawal threshold for that trial. The trial was repeated once again for the left hind paw, with monofilaments applied in ascending grams force until a positive response. Next, the second mouse on the apparatus was tested for two trials on left hind paw, followed by third mouse in the apparatus and the fourth. Then, the same two‐trial procedure was repeated for each mouse using the plantar surface of each right paw. Averaged paw withdrawal thresholds are reported (Neurobehavioral and [JAX‐MNBF], 2018) Thermal sensitivity was assessed with the hot plate test, performed as previously described (Hermanson et al., 2013), with mice placed onto a flat surface preheated to 50°C within an open Plexiglas tube. Latency to the first positive response was recorded, with a cutoff time of 40 s. Positive responses included hind paw shaking or hind paw licking, or mouse jumping. Latency in seconds to positive response or reaching cutoff time with no positive responses for one trial per mouse is reported.

### Irritability‐like behavior tests

To measure “irritability,” we used the bottlebrush test for mice (Kononoff et al., 2018; Riittinen et al., 1986). After 30 min of acclimation to the testing room, mice were placed alone in their open home cage. Two cameras were aimed at the clear cage: one from a lateral side view and one overhead. The experimenter used a bottlebrush—a small white plastic brushed with a rounded top of protruding plastic bristles, used to clean small bottles. Briefly, mice were lightly “attacked” by the brush (touched by the bristles of the brush and followed around by the brush) in the following sequence:

1. Brush (in rotation) approaching the mouse from the end (starting position) of the cage.
2. Brush (in rotation) touching the whiskers of the mouse.
3. Brush (in rotation) returning to the starting position in the opposite end of the.
4. Brush (in rotation) at the starting position.
5. Brush (no rotation) at the starting position.

Each mouse was attacked by the bottlebrush 20 times per session. Videos were coded using ANVIL video coding software for the following positive responses: (1) Mouse was climbing cage wall during the bottle brush attack, (2) digging, (3) running away/escaping the bottlebrush, (4) freezing in response to the bottlebrush attack, or (5) exploring the bottlebrush as it was touching them. Using the ANVIL time coding function, the duration of each positive response was recorded. The total time for each positive response during 20 bottlebrush attacks is reported for each mouse.

### Anxiety‐like behavior tests

Elevated plus‐maze (EPM), open field test (OFT), and light–dark box (LD) were performed exactly as previously described (Morgan et al., 2019).

The EPM had two wall‐free open‐arms (30 × 10 cm; light illuminance ~100–115 lux), and two walled (“closed”) arms (30 × 10 × 15 cm; ~20–25 lux) anchored to a square 5 × 5 cm open center. To begin the test and trigger video tracking, mice were placed in the center, facing an open arm. Maze was made of white acrylonitrile butadiene styrene plastic and elevated 47 cm off the ground. Each mouse explored the EPM for 5 min per test, with ANY‐maze (Stoelting, Wood Dale, Illinois) video tracking software used to monitor and analyze behavior during testing.

The OFT used a square sound‐attenuating chamber with clear plexiglass walls (27.9 × 27.9 × 20.3 cm; MED‐OFA‐510; MED Associates, St. Albans, Vermont) contained within a white sound‐attenuating chamber box. Mice were placed in the center of the open field chamber to begin the test and recorded for 5 min, with beam breaks from 16 infrared light beams measuring movement and position with Activity Monitor v5.10 (MED Associates) software. The center of the OFT was designated as a square of the innermost 50% of the OFT arena floor, with the remainder considered the perimeter. The chamber was illuminated at ~200 lux and white noise was present at ~60 dB.

The LD box test used a black insert (Med Associates ENV‐511; made of IRT to allow infrared beam transmission) was placed into a chamber with clear plexiglass walls (27.9 × 27.9 × 20.3 cm; MED‐OFA‐510) to split the chamber into equal halves light (~350–400 lux) and dark (<5 lux) divisions. Beam breaks from 16 infrared beams were recorded in Activity Monitor v5.10 (MED Associates) to monitor position and behavior during the 10‐min test. White noise was present at ~60 dB.

## RESULTS

### Mechanical hypersensitivity is associated with EtOH withdrawal

**FIGURE 1:**
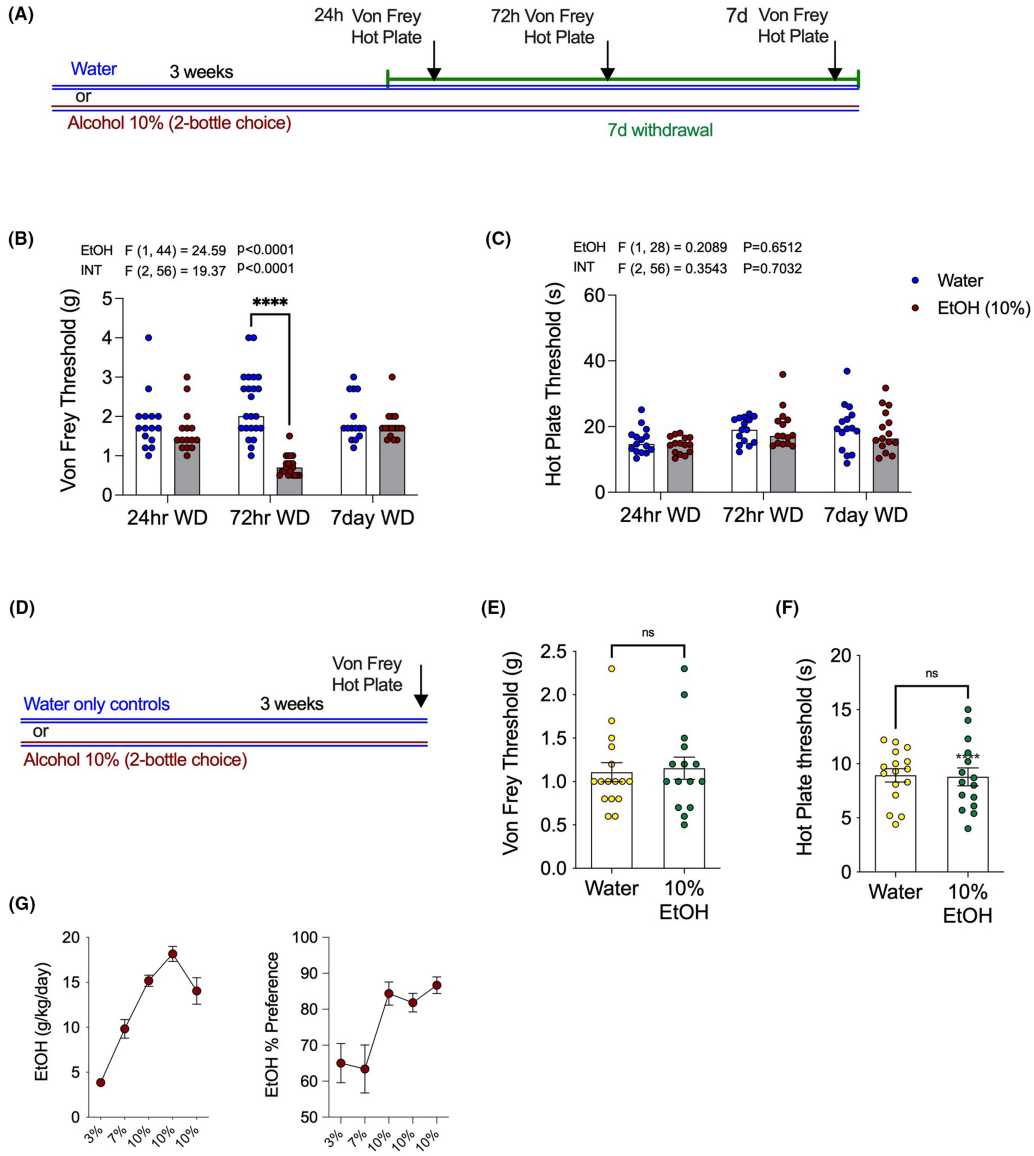
Alcohol withdrawal produces transient mechanical but not thermal hypersensitivity. (A) Schematic of 21‐day 2BC EtOH drinking paradigm and behavioral testing at withdrawal time points. (B) Mechanical hypersensitivity is seen only at the 72 h EtOH withdrawal time point, with reduced Von Frey threshold compared with water drinking controls. (C) No effects on thermal sensitivity were observed at any withdrawal time point measured using the hot plate test. (D) Schematic of behavioral testing during drinking 10% EtOH or water‐only. No effect on (E) Von Frey or (F) Hot plate threshold was observed during alcohol drinking. (G) Weekly EtOH intake and preference of alcohol‐drinking mice for graphs B and C. *F*‐scores on graphs represent main effects or interactions from two‐way ANOVAs. ****p < 0.0001 via post hoc Holm–Sidak pairwise comparisons, NS; not significant. Data presented as individual female mice and Mean ± SEM, with number of subjects per group as follows: Graphs B/C/G water‐only *n* = 15–23, EtOH drinking *n* = 15–23; graphs E/F water‐only *n* = 16, EtOH drinking *n* = 15.

We first investigated the effects of withdrawal from 10% EtOH on nocifensive behaviors (Figure 1). Mechanical and thermal sensitivity were measured 24 h, 72 h, and 1 week into withdrawal (Figure 1A). Compared with water drinking controls, EtOH withdrawal mice showed increased mechanical sensitivity, with significantly lower paw withdrawal thresholds on the Von Frey filament test at 72 h into withdrawal. However, no significant effect of EtOH withdrawal was seen at the earlier withdrawal time point of 24 h, or at the late time point of one‐week post‐EtOH withdrawal (Figure 1B). By contrast, there was no difference between alcohol withdrawal and water drinking controls on the hot plate test of thermal sensitivity at any time point (Figure 1C). A separate cohort of mice were tested on the Von Frey filament and hot plate tests after three weeks stable drinking on 10% EtOH in 2BC, that is, not in withdrawal (Figure 1D). Mice drinking 10% EtOH in 2BC were not different from water controls in terms of either Von Frey (Figure 1E) or hot plate (Figure 1F) thresholds. These data indicate that alcohol withdrawal is associated with a timed‐dependent emergence of mechanical, but not thermal, hypersensitivity, and that this effect is not observed during alcohol drinking per se, and thus selective to the withdrawal state.

### JZL184 reverses mechanical hypersensitivity associated with EtOH withdrawal

**FIGURE 2:**
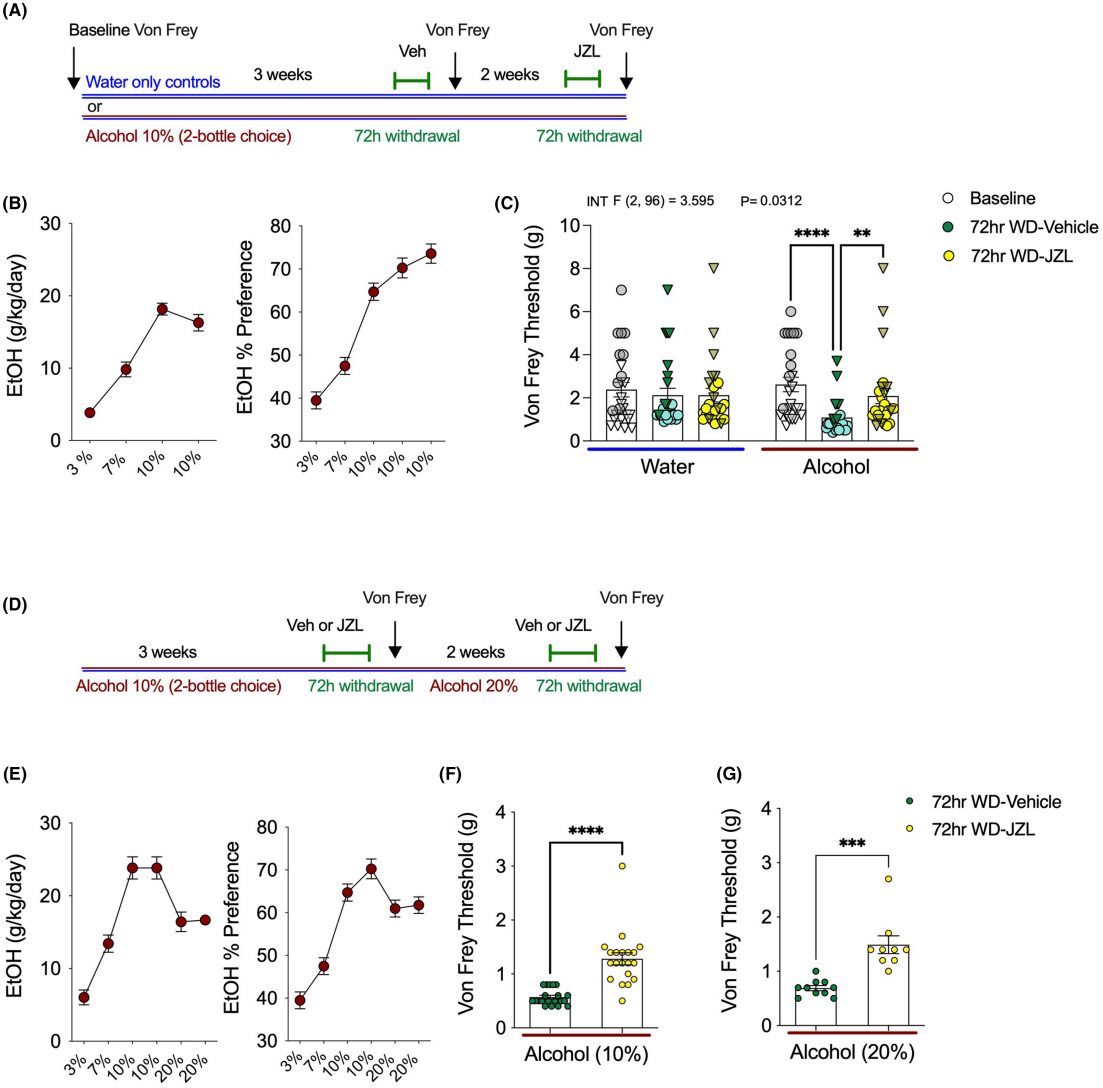
JZL184 reverses mechanical hypersensitivity 72 h into EtOH withdrawal. (A) Schematic of experimental timeline. (B) Weekly EtOH intake of alcohol‐drinking mice. (C) Von Frey threshold is significantly reduced at 72 h into EtOH withdrawal, which is reversed by JZL184. (D) Schematic experimental timeline. (E) Weekly EtOH intake of alcohol‐drinking mice. (F and G) JZL184 reverses mechanical hypersensitivity at 72 h EtOH withdrawal from 10% and 20% EtOH. *F* and *p* values shown in figures from two‐way ANOVA. **p < 0.01, ***p < 0.001, ****p < 0.0001 via post hoc Holm–Sidak pairwise comparisons or unpaired two‐tailed *t*‐test. Data presented as individual mice (triangles males and circles females) and Mean ± SEM, with number of subjects per group as follows: Graphs B/C water‐only *n* = 26, EtOH drinking *n* = 25; graphs E–G 10% EtOH drinking vehicle treatment *n* = 20, 10% EtOH drinking JZL184 treatment *n* = 20. 20% EtOH drinking vehicle treatment *n* = 10, 20% EtOH drinking JZL184 treatment *n* = 9.

We next conducted several independent experiments to determine whether pharmacologically increasing 2‐AG levels affected the increased mechanical sensitivity observed during EtOH withdrawal. First, baseline Von Frey threshold was determined prior to initiation of the 2BC model (Figure 2A). After ramping up from 3% to 10% EtOH, mice drank 10% EtOH for three weeks and were tested using the Von Frey assay at 72 h into withdrawal (Figure 2A). EtOH withdrawn mice treated with vehicle had significantly reduced Von Frey thresholds compared with their own baselines, whereas no changes in Von Frey threshold were detected in water drinking control mice across these time points (Figure 2C). Beginning immediately after 72 hr withdrawal Von Frey testing was completed, the EtOH mice were placed back on 2BC 10% EtOH for two additional weeks. After two weeks of additional drinking, EtOH was again removed for mechanical sensitivity testing at 72 h withdrawal. This time, water mice and EtOH mice were pretreated with MAGL inhibitor JZL184 (10 mg kg^−1^) to determine whether increasing 2‐AG levels could reverse hyperalgesia at 72 h of EtOH withdrawal, and whether JZL184 has analgesic properties in control mice drinking water only (Figure 2A). JZL184 normalized mechanical hypersensitivity by significantly increasing Von Frey thresholds in mice in EtOH withdrawal (Figure 2C). The water‐only group tested alongside the withdrawal mice showed no differences when tested at baseline, with vehicle, or with JZL184 pretreatment.

To confirm these findings using a between‐subjects design, a separate cohort of 2BC EtOH drinking mice were treated with vehicle or JZL184 (10 mg kg^−1^) and tested at 72 h withdrawal from 10% EtOH (Figure 2D). Consistent with our previous experiment, JZL184 was able to significantly increase Von Frey thresholds relative to vehicle treatment at 72 h into EtOH withdrawal (Figure 2F). Immediately after Von Frey testing with vehicle or JZL184 pretreatment, mice were put back into 2BC cages and given one bottle of water and one bottle of 20% EtOH. After 2 weeks of drinking, access to 20% EtOH was removed, and Von Frey testing was again conducted at 72 h into withdrawal (Figure 2D). Again, 72 h after access to 20% EtOH was removed, mechanical hypersensitivity was seen in vehicle‐treated mice (Figure 2G). There were no differences in the hyperalgesia triggered by removal of access from 20%, relative to 10%, EtOH (Figure 2F vs. 2G). Again, confirming our previous experiment, JZL184 was also able to reverse mechanical hypersensitivity 72 h into withdrawal from 20% EtOH (Figure 2G). These data provide compelling evidence that MAGL inhibition can reduce mechanical hypersensitivity induced by alcohol withdrawal.

### Hyperalgesia during EtOH withdrawal is not associated with concurrent increases in anxiety‐like behavior

**FIGURE 3:**
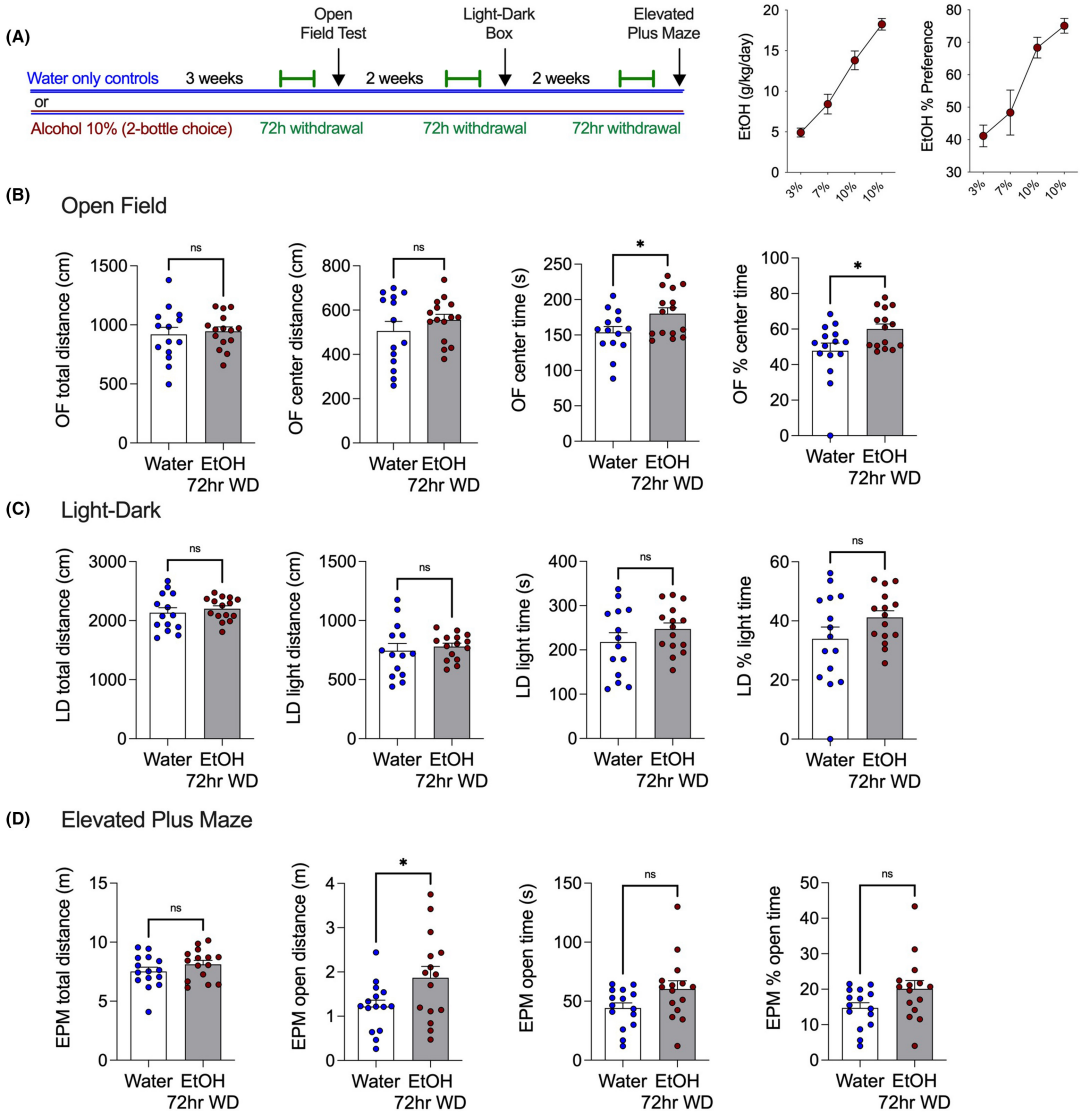
EtOH withdrawal does not increase anxiety‐like behaviors. (A) Schematic of experimental timeline and weekly EtOH intake and preference of alcohol‐drinking mice. (B) Effects of 72 h EtOH withdrawal on open field test. (C) Effects of 72 h EtOH withdrawal on light–dark test. (D) Effects of 72 h EtOH withdrawal on elevated plus‐maze. *p < 0.05, via unpaired two‐tailed *t*‐test. Data presented as individual female mice and Mean ± SEM, with number of subjects per group as follows: Graphs B water‐only *n* = 14, EtOH drinking *n* = 15; graphs C water‐only *n* = 14, EtOH drinking *n* = 15. Graphs D water‐only *n* = 15, EtOH drinking *n* = 15.

We considered the possibility that EtOH withdrawal‐induced hyperalgesia could be considered one effect among several seen in the broader context of a general aversive behavioral response to alcohol withdrawal. To determine whether mechanical hypersensitivity was an independent effect of EtOH withdrawal or was associated with an increase in anxiety‐like behavior, the open field test was carried out during 72 h withdrawal with a new cohort of 10% EtOH drinking mice. The EtOH withdrawal mice were no different in the total or center distance traveled in the open field test but spent more time in the center of the apparatus than water drinking controls (Figure 3). After this, the light–dark box and elevated plus‐maze tests were conducted after mice were put back on EtOH for 2 weeks to achieve stable drinking levels, and then EtOH was removed for 72 h (Figure 3A). Mice in EtOH withdrawal did not exhibit increase anxiety‐like behavior on any measure. The lack of differences between groups indicates the mechanical hypersensitivity seen in EtOH withdrawal groups is not associated with increases in anxiety‐like behaviors at the 72‐h time point.

### EtOH withdrawal induces irritability behavior, which is not prevented by MAGL inhibition

**FIGURE 4:**
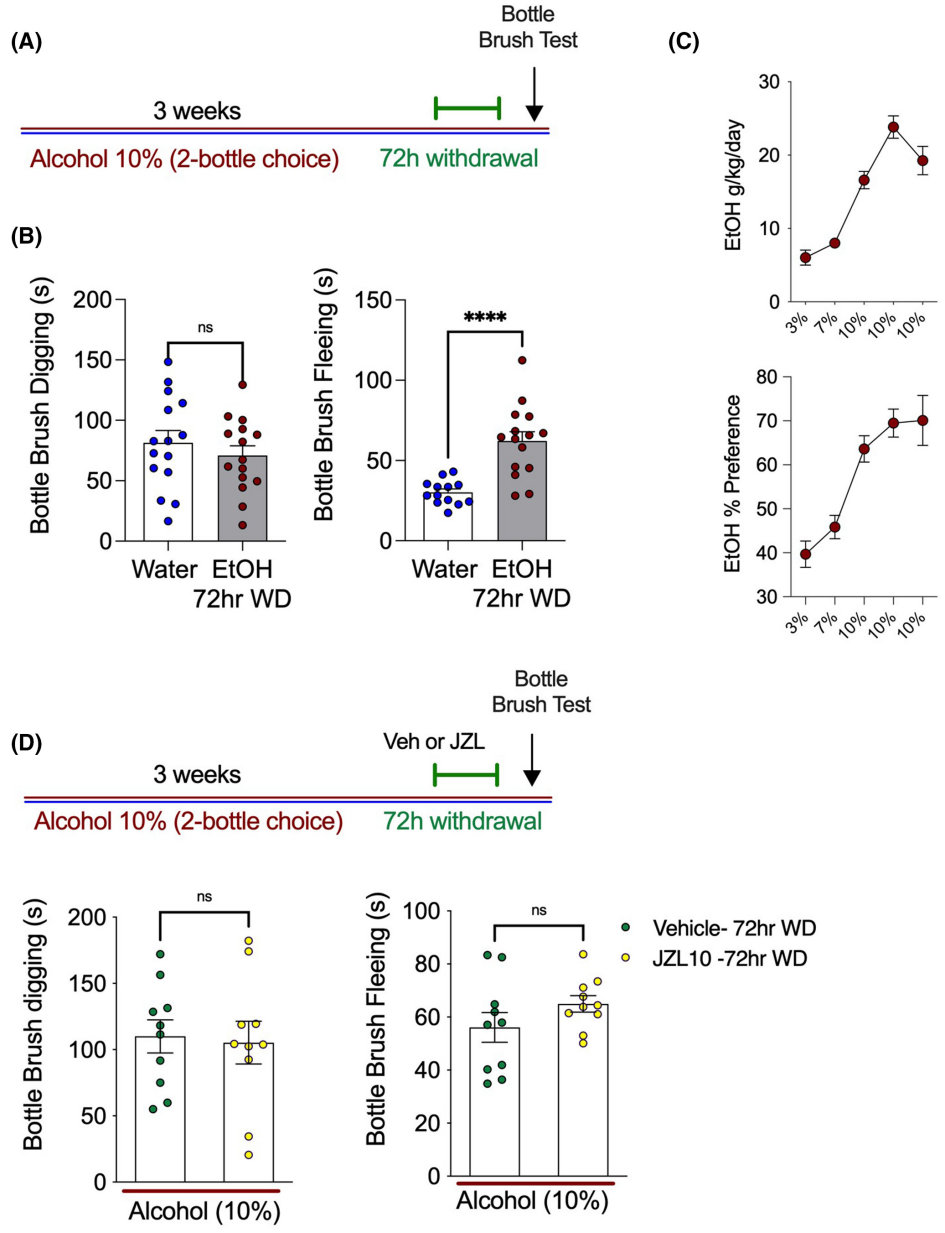
EtOH withdrawal increases irritability‐like behavior, which is not prevented by JZL184. (A) Schematic of 2BC drinking with irritability‐like behavior tested at 72 h of EtOH withdrawal. (B) 72‐h EtOH withdrawal had no effect on digging behavior in the bottlebrush test (left), but significantly increased fleeing behavior (right). (C) Weekly EtOH intake of alcohol‐drinking mice for graph (B). (D) A separate cohort was tested for irritability at 72 h withdrawal and given vehicle or JZL184. (Left) JZL184 had no effect on digging or fleeing in the bottlebrush test. ****p < 0.0001 via unpaired two‐tailed *t*‐test. Data presented as individual female mice and Mean ± SEM, with number of subjects per group as follows: Graphs B/C water‐only *n* = 15, EtOH drinking *n* = 15; graphs C water‐only *n* = 14, EtOH drinking *n* = 15. Graph D EtOH drinking vehicle treatment *n* = 10, EtOH drinking JZL184 treatment *n* = 10.

In the next experiment, mice in EtOH withdrawal were tested to determine whether they expressed concurrent states of irritability‐like behavior measured using the bottlebrush test during EtOH withdrawal. Compared with water‐only controls, EtOH withdrawal mice engaged in fleeing, but not digging, behavior significantly more often in the bottlebrush irritability test (Figure 4A). Furthermore, treatment with JZL184 (10 mg kg^−1^) was not able to prevent increases in irritability‐like behavior in a separate cohort of EtOH drinking mice in 72 h withdrawal (Figure 4B). EtOH withdrawal thus results in irritability and mechanical hypersensitivity. This lack of sensitivity to JZL184 suggests the neural mechanisms underlying mechanical hypersensitivity triggered by EtOH withdrawal and irritability‐like behaviors are likely dissociable.

### Both CB_1_ and CB_2_ receptor antagonists prevent the antihyperalgesic effects of MAGL inhibition and exacerbate mechanical hypersensitivity during EtOH withdrawal

**FIGURE 5:**
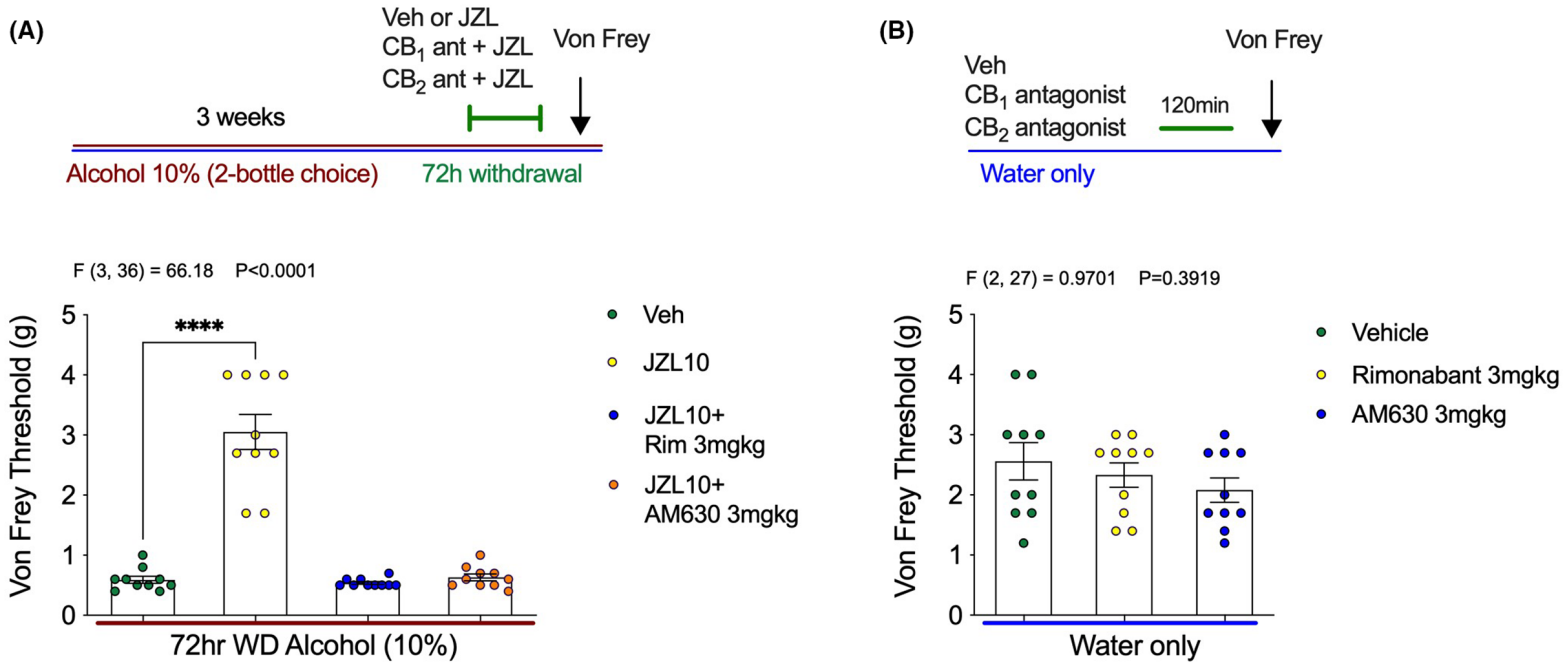
CB_1_ and CB_2_ receptor antagonists prevent the antihyperalgesic effects of JZL184. (A) Both Rimonabant and AM630 prevent the antihyperalgesic effects of JZL184. (B) Neither Rimonabant nor AM630 affect mechanical sensitivity thresholds in water drinking mice. *F* and *p* values shown in figures from one‐way ANOVA. ****p < 0.0001 via post hoc Holm–Sidak pairwise comparisons. Data presented as individual female mice and Mean ± SEM with number of subjects per group as follows: Graph A *n* = 10 per group, graph B *n* = 10 per group.

Because JZL184 reverses mechanical hypersensitivity during EtOH withdrawal, we next sought to further characterize the pharmacological mechanisms underlying these effects. To determine whether the antihyperalgesic properties of JZL184 were mediated through CB_1_ or CB_2_ receptors, mice underwent 72 h of EtOH withdrawal and were treated with JZL184 alone, or JZL184 in combination with CB_1_ or CB_2_ antagonists (Figure 5). Mechanical hypersensitivity returned when JZL184 was co‐administered with either the CB_1_ antagonist Rimonabant (3 mg kg^−1^) or the CB_2_ antagonist AM630 (3 mg kg^−1^). The antinociceptive effect of JZL184 was completely blocked by both CB receptor antagonists (Figure 5A). No differences in Von Frey threshold were seen in control mice that only drank water and were treated with Rimonabant (3 mg kg^−1^), or AM630 (3 mg kg^−1^), 2 h before the Von Frey testing (Figure 5B). These data support the involvement of CB_1_ and CB_2_ receptors in the antihyperalgesic effects of JZL184.

### The DAGL inhibitor DO34 enhanced mechanical hypersensitivity during EtOH withdrawal

**FIGURE 6:**
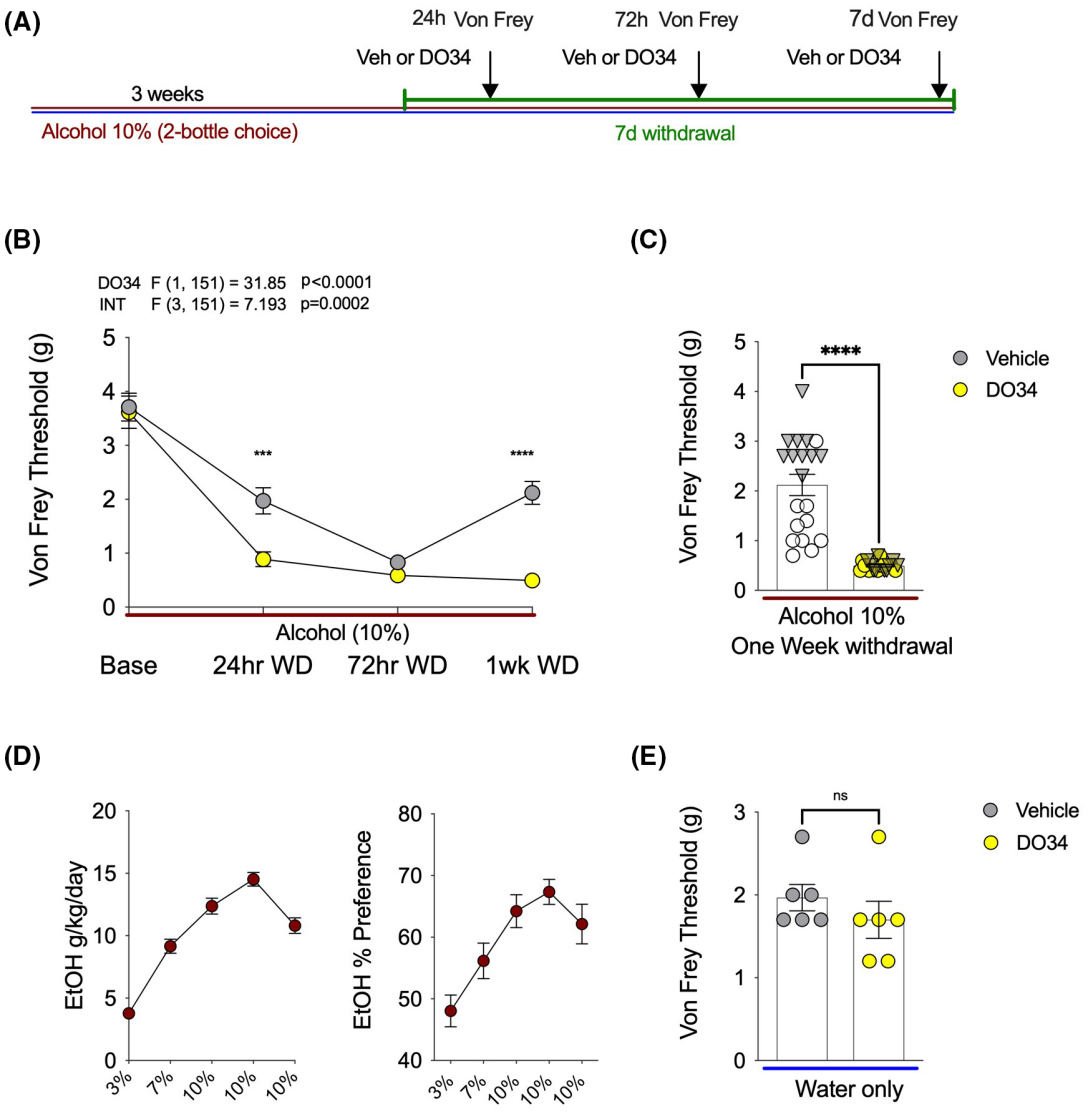
DO34 exacerbates mechanical hypersensitivity during EtOH withdrawal. (A) Schematic of experimental timeline and drug treatments. (B) DO34 decreased Von Frey threshold at 24 h and one‐week into EtOH withdrawal relative to vehicle‐treated mice. (C) The same results as 1 week withdrawal separating males (circles) and female (triangles) data points for visual representation of data distribution. (D) Weekly EtOH intake and preference for mice shown in (B and C). (E) D034 does not affect sensitivity thresholds in water‐only drinking mice. *F* and *p* values on graphs represent main effects and interaction from two‐way ANOVA. ***p < 0.001, ****p < 0.0001 via post hoc Holm–Sidak pairwise comparisons or unpaired *t*‐test. NS; not significant. Data presented as individual mice (triangles males and circles females) and Mean ± SEM, number of subjects per group as follows: Graphs B/C/D *n* = 20 per group (72 hr withdrawal DO34 treatment *n* = 19). Graph E *n* = 6 per group.

The lowered mechanical sensitivity threshold triggered by 72 EtOH withdrawal was prevented with a MAGL inhibitor, and this effect was blocked by both CB_1_ and CB_2_ antagonists. To further characterize the specific role of 2‐AG signaling in the regulation of mechanical hypersensitivity during EtOH withdrawal, the DAGL inhibitor DO34 was used to block the endogenous synthesis of 2‐AG (Figure 6A). After 3 weeks of 2BC drinking, EtOH bottles were removed, and the Von Frey testing was completed after 24 h, 72 h, and 1 week of withdrawal. DO34 pretreatment 2 h prior to each testing time point led to a robust emergence of previously unseen mechanical hypersensitivity at 24 h withdrawal, with significantly lowered Von Frey thresholds (Figure 6B). Next, as predicted, the mechanical thresholds of both vehicle and DO34 groups were very low on the Von Frey test at 72 h withdrawal. Finally, DO34 completely prevented recovery of normal mechanical sensitivity responses and caused persistent withdrawal‐triggered hypersensitivity at 1 week into EtOH withdrawal (Figure 6B,C). Alcohol consumption and preference for this cohort of mice are shown in Figure 6D. Lastly, we showed that DO34 did not affect mechanical sensitivity thresholds in water drinking mice (Figure 6E). These data indicate that inhibition of DAGL leads to an earlier onset and persistent mechanical hypersensitivity induced by EtOH withdrawal, and that the effects of 2‐AG depletion are specific to the alcohol withdrawal state as DO34 has no effect on sensitivity thresholds in control mice.

**FIGURE 7:**
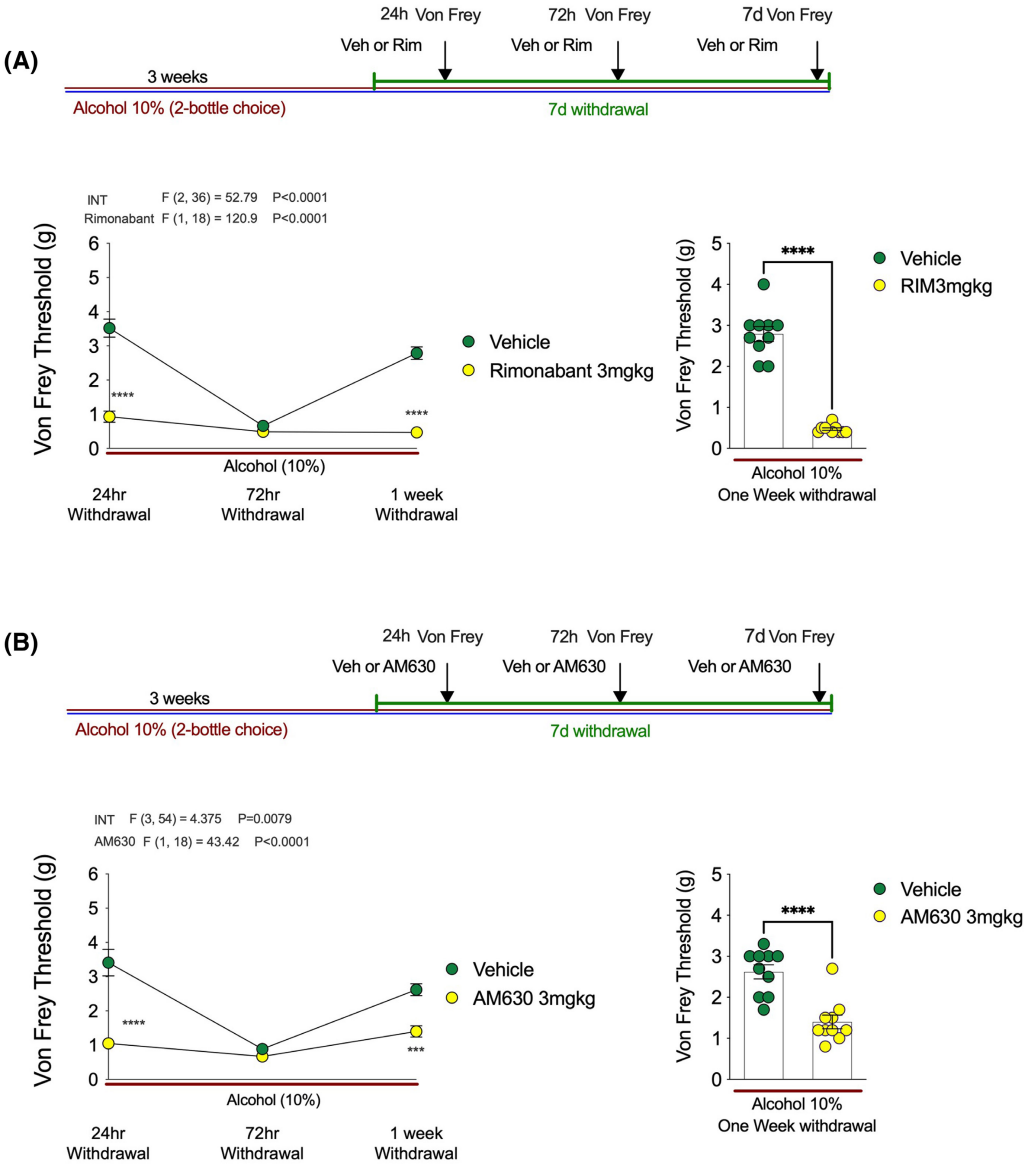
CB_1_ and CB_2_ receptor blockade enhance alcohol withdrawal‐induced mechanical hypersensitivity. (A) Rimonabant decreased Von Frey threshold at 24 h and one‐week into EtOH withdrawal relative to vehicle‐treated mice. (B) AM630 decreased Von Frey threshold at 24 h and one‐week into EtOH withdrawal relative to vehicle‐treated mice. Bar graphs in (A) and (B) depict 1 week data for visual depiction of data distribution. *F* and *p* values on graphs represent main effects and interaction from two‐way ANOVA. ***p < 0.001, ****p < 0.0001 via post hoc Holm–Sidak pairwise comparisons or unpaired *t*‐test. Data presented as individual mice and Mean ± SEM, number of subjects per group as follows: Graphs A/B *n* = 10 per group.

Given that both CB_1_ and CB_2_ receptors were implicated in the antihyperalgesic effects of JZL184, we next tested whether CB_1_ or CB_2_ receptor blockade could enhance alcohol withdrawal‐induced mechanical hypersensitivity (Figure 7). Following the same experimental design as for our DAGL inhibition experiments, we showed that blocking CB_1_ receptors with Rimonabant increased mechanical sensitivity at 24 h and 1 week postwithdrawal in a manner identical to DO34 (Figure 7A). Similarly, blockade of the CB_2_ receptor increased mechanical sensitivity at 24 h and 1 week postwithdrawal (Figure 7B). These data indicate that endogenous 2‐AG counteracts alcohol withdrawal‐associated mechanical hypersensitivity via action at both CB_1_ and CB_2_ receptors, and in the absence of this signaling system withdrawal‐induced hyperalgesia is significantly exacerbated and prolonged.

## DISCUSSION

Here, we show that pharmacological inhibition of MAGL with JZL184 prevents alcohol withdrawal‐induced mechanical hypersensitivity via activation of CB_1_ and CB_2_ receptors. Importantly, neither 2‐AG augmentation affected mechanical sensitivity thresholds in water drinking mice or in mice exposed to alcohol but not in withdrawal. These data are partially consistent with recent data indicating MAGL inhibition within the lateral habenula reduces mechanical hypersensitivity in rats undergoing alcohol withdrawal; however, in that study, MAGL inhibition also increased mechanical thresholds to a similar degree in control rats (Fu et al., 2021). We also showed that endogenous 2‐AG acting via CB_1_ and CB_2_ receptors serves to counteract alcohol withdrawal‐associated mechanical hypersensitivity, as blockade of 2‐AG synthesis (and blockade of either CB_1_ or CB_2_ receptors) accelerated the onset and dramatically extended the time course of hyperalgesia in our model. That blockade of 2‐AG synthesis had no effect on mechanical sensitivity thresholds in control mice suggests alcohol withdrawal is associated with an adapted state during which endogenous 2‐AG exerts analgesic effects to limit the adverse consequences of alcohol withdrawal on pain sensitivity. These data demonstrate a critical role for 2‐AG in the bidirectional modulation of mechanical sensitivity thresholds selectively during alcohol withdrawal and suggest MAGL inhibition could represent a novel approach to the treatment of hyperalgesic states associated with alcohol withdrawal. These data identify 2‐AG‐mediated eCB signaling as a key physiological signaling system limiting the duration of alcohol withdrawal‐associated mechanical hypersensitivity.

Monoacylglycerol lipase inhibition has been shown to reduce neuropathic pain, chemotherapy‐induced neuropathic pain (Curry et al., 2018), pain induced by models of multiple sclerosis (Brindisi et al., 2016), pain arising from inflammatory states (Burston et al., 2016), joint pain in osteoarthritis models (Philpott & McDougall, 2020), and chronic stress‐induced hyperalgesia (Lomazzo et al., 2015). Previous studies have also implicated CB_1_ and CB_2_ receptors in the analgesic effects of MAGL inhibition in various preclinical models (Ghosh et al., 2013; Thomas et al., 2020). Our data and a recent study (Fu et al., 2021) confirm the preclinical efficacy of MAGL inhibition in the reduction of mechanical hypersensitivity associated with alcohol withdrawal. Importantly, evidence that MAGL inhibition can increase hedonic behavior (Gianessi et al., 2019); however, we have previously shown that MAGL inhibition did not increase alcohol consumption or preference in mice (Winters et al., 2021), and direct lateral habenula MAGL inhibition actually reduced operant alcohol self‐administration (Fu et al., 2021). Moreover, MAGL inhibition has been shown to reduce negative affective states induced by alcohol withdrawal (Holleran et al., 2016). These findings, combined with our present results, suggest MAGL inhibition could represent an important new approach to reduce negative reinforcement driven alcohol use by reducing both negative affective and somatic states associated with alcohol withdrawal without propensity to increase alcohol intake per se.

The present studies also support the critical role of endogenous 2‐AG signaling in pain processing during alcohol withdrawal. Postsynaptic 2‐AG is synthesized on‐demand by the enzyme DAGL⍺ in neurons (Bisogno et al., 2003; Shonesy et al., 2014). Pharmacological inhibition of DAGL via the compound DO34 results in dramatic decreases in brain 2‐AG levels (Ogasawara et al., 2016; Winters et al., 2021). DAGL⍺^−/−^ mice exhibit more anxiety‐like and depression‐like behaviors at baseline (Jenniches et al., 2016; Shonesy et al., 2014), and DO34 50 mg kg^−1^ treatment after acute stress increases anxiety and depression‐like behaviors (Bluett et al., 2017) and impairs fear extinction in mice (Cavener et al., 2018). The current studies demonstrate that blocking synthesis of 2‐AG exacerbates pain sensitivity, causing an earlier onset of mechanical hypersensitivity during withdrawal (24 h), which persisted beyond the recovery time established in the previous experiments. Under the current experimental conditions, mice treated with DO34 demonstrated robust mechanical hypersensitivity that persisted 7 days into alcohol withdrawal. The current results are difficult to directly compare with previous findings, with one published study by Wilkerson et al. (2017) indicating pain sensitivity was reduced by DO34 in a model of LPS‐induced allodynia. However, this could be due in part to anti‐inflammatory effects secondary to reduced production of 2‐AG‐derived pro‐inflammatory prostaglandins. We have shown previously that 2‐AG‐derived pro‐inflammatory prostaglandin glycerol esters (PG‐Gs) can be generated following LPS‐driven upregulation of cyclooxygenase‐2 (Morgan et al., 2018), and several other reports have shown LPS increases the production of pro‐inflammatory prostaglandins (Britt et al., 2012). Future studies will be needed to determine the specific conditions under which 2‐AG signaling represents an antihyperalgesic system.

Here, we highlight the role of 2‐AG signaling in pain sensitivity during EtOH withdrawal, but some questions remain unanswered. The mechanical hypersensitivity seen during EtOH withdrawal is exacerbated by DO34, suggesting deficiencies in 2‐AG signaling may be a susceptibility endophenotype for the development of increased pain sensitivity during withdrawal. In the context of AUD, the increased pain sensitivity seen during alcohol withdrawal presents a barrier to prevent relapse in clinical populations. We recently reported that inhibiting DAGL reduces EtOH drinking and re‐initiation in mice (Winters et al., 2021). It is possible that the anticraving effects of DAGL inhibition override the potential increase in motivation to seek alcohol driven by the enhanced hyperalgesic state. Future studies will be required to test these hypotheses and elucidate neural mechanisms subserving these effects. Additionally, more research is needed on the interactions of eCBs with current therapeutics such as Gabapentin, which is used to treat both chronic pain and alcohol use disorder (Kranzler et al., 2019). Little is known about the role of eCBs in Gabapentin‐mediated reduction in alcohol consumption. Roberto et al. (2008) report Gabapentin modulates receptor‐mediated IPSCs (GABA‐IPSCs) in CeA neurons in alcohol‐dependent rats. Behaviorally, Gabapentin reduced EtOH intake and prevented negative affective behaviors during withdrawal, and when infused directly into the CeA, prevented dependence‐induced elevation in operant EtOHresponding (Roberto et al., 2008). Despite ample evidence of the analgesic properties of Gabapentin, few studies have examined how eCBs may interact with Gabapentin to treat acute or chronic pain (Wiffen et al., 2017). One paper by Crowe et al. (2017) reported that Gabapentin co‐administered with a MAGL inhibitor synergistically improved allodynia in a model of neuropathic pain in mice. This paper also reported Gabapentin elevated Prostaglandin E2 (PGE_2_) in the spinal cord (Crowe et al., 2017), and 2‐AG is thought to be the major source of AA for PG production in the CNS (Nomura et al., 2011). The mechanisms underlying the interactions between MAGL inhibition and currently available treatments represents another important area of investigation for future studies.

Previous studies have implicated CB_1_ receptors in the suppression of nociceptive transmission (Hohmann et al., 1995; Kelly & Chapman, 2001). For example, Rimonabant administration results in hyperalgesia in the formalin paw test (Strangman et al., 1998) and hot plate test (Richardson et al., 1998), and reverses stress‐induced analgesia (Hohmann et al., 2005). Taken together, these data suggest alcohol withdrawal recruits 2‐AG signaling to counteract mechanical hypersensitivity over extended time scales (at least ~1 week). Our data also indicate that blockade of CB_1_ or CB_2_ receptors also prolongs alcohol withdrawal‐associated hyperalgesia. One explanation for these data is that 2‐AG signaling is enhanced within distinct neuronal circuits and acts at CB_1_ and CB_2_ receptors to limit the duration and magnitude of withdrawal‐associated hyperalgesia. Indeed, the mechanical hypersensitivity seen here at 72 h EtOH withdrawal is a relatively brief effect, with vehicle‐treated groups returning to baseline sensitivity after 7 days without EtOH access. Our present data indicate the reason for the limited duration of hyperalgesia is 2‐AG‐mediated buffering of this response, as inhibition of 2‐AG‐mediated eCB signaling produced dramatically prolonged hyperalgesic states in mice. It is possible that withdrawal from more severe forms of alcohol exposure could override this buffering system and result in prolonged hyperalgesia; however, this hypothesis remains to be tested.

In clinical literature, irritability is one of three conditions contributing to a negative emotional states during drug withdrawal (dysphoria, anxiety, and irritability) (Koob & Le Moal, 2001) and contributing to relapse (Koob & Le Moal, 2005). Irritability‐like behavior is challenging to model in rodents, with early alcohol withdrawal experiments relying on subjective experimenter observations of “irritability” in rodents (Ulrichsen et al., 1986). The bottlebrush test used here is a measure of irritability in rodents based on a tactile stimulus, which has been shown to detected the presence of irritability‐like behavior during alcohol withdrawal (Kimbrough et al., 2017) and oxycodone withdrawal (de Guglielmo et al., 2019). It is possible that the decreased withdrawal thresholds (increased mechanical sensitivity) measured by the Von Frey tests could be related to irritability rather than hyperalgesia per se. Indeed, we did detect increases in irritability‐like behavior at 72 h of withdrawal; however, this effect was not reversed by JZL184 treatment. These data suggest that reduced withdrawal thresholds observed using the Von Frey test were not likely secondary to changes in irritability, since they were robustly reversed by JZL184, whereas irritability‐like behaviors were not affected by JZL184.

There are some limitations to the present work which should be considered when interpreting our findings. For example, the pharmacological approaches utilized here are systemic, and therefore, the actions of 2‐AG during EtOH withdrawal are not localized to specific brain regions or circuits. Thus, the use of pharmacological tools alone is limited and should be complemented by genetic approaches and site‐specific infusions in future studies. Additionally, further work is required to determine whether these effects are sex‐specific. Female C57BL/6J mice were used primarily in these experiments, due their increased EtOH consumption and preference, compared with male mice (Centanni et al., 2019; Winters et al., 2021). Female rodents are also more sensitive to the analgesic and antinociceptive properties of cannabinoids and endocannabinoids (Blanton et al., 2021). There is little preclinical evidence to suggest sex differences in EtOH withdrawal‐induced pain thresholds, but clinical studies do indicate females are more responsive to painful stimuli, have a lower pain threshold, and more likely to report chronic pain (Sorge & Totsch, 2017). Future studies should be aimed at explicitly testing for sex differences in eCB modulation of withdrawal‐induced hyperalgesia.

In conclusion, here, we show that withdrawal‐induced mechanical hypersensitivity emerges 72 h into alcohol withdrawal from a 2BC paradigm, and this effect is reversed by JZL184 in male and female mice. The antihyperalgesic effects of MAGL inhibition are mediated through CB_1_ and CB_2_ receptors. Importantly, inhibition of DAGL, or blockade of CB_1_ or CB_2_ receptors, exacerbates and prolongs mechanical hypersensitivity. These data suggest 2‐AG signaling is a key mechanism recruited during alcohol withdrawal to buffer against the development of hyperalgesia and that pharmacological augmentation of 2‐AG levels could represent a novel treatment for pain associated with alcohol withdrawal and patients with AUD‐pain comorbidity.

## AUTHOR CONTRIBUTIONS

AM and SP designed experiments and wrote the manuscript. AM performed experiments and analyzed data. DA, KJ, and EB performed experiments and maintained mouse EtOH colonies.

## CONFLICT OF INTEREST

S.P. is a scientific consultant for Psy Therapeutics and Jazz Pharmaceuticals. The remaining authors have nothing to disclose.

## Supporting information


Appendix S1
