# Supplementary material for: 2‐Arachidonoylglycerol‐mediated endocannabinoid signaling modulates mechanical hypersensitivity associated with alcohol withdrawal in mice
Source: Alcohol Clin Exp Res. 2022 Oct 12;46(11):2010–24. doi: 10.1111/acer.14949 (PMC10091740; doi:10.1111/acer.14949)
Supplement: Supplementary file 1 — Appendix S1 [file ACER-46-2010-s001.docx]

**Supplemental Information**

**Role of CB_1_ and CB_2_ receptors in nociception during EtOH withdrawal**

Augmentation of 2-AG levels via pharmacological MAGL inhibition has been shown to act on both CB_1_ and CB_2_ receptors to reduce pain sensitivity (Ghosh et al., 2013, Ignatowska-Jankowska et al., 2015, Curry et al., 2018, Jiang et al., 2015, Thomas et al., 2020). In the brain, the CB_1_ receptor is the most dominant eCB binding site (Elphick and Egertova, 2001, Patel et al., 2017), while the CB_2_ receptor is largely associated with immune cells and microglia in the CNS (Patel et al., 2017, Atwood et al., 2012, Fernández-Ruiz et al., 2007, Parolaro, 1999). Our pharmacological studies demonstrate blockade of both CB_1_ and CB_2_ receptors prevents the anti- antinociceptive effects of JZL184 during EtOH withdrawal. These data indicate JZL184 given during alcohol withdrawal reduces mechanical hypersensitivity through activation of both CB_1_ and CB_2_ receptors, consistent with a dual receptor mechanism in a variety of other models noted above. The relative contribution of central vs. peripheral cannabinoid receptors to the anti-hyperalgesic effects of JZL184 during alcohol withdrawal is not known but is an important area for future investigation that could have important implications for 2-AG-based therapeutics development.

**Type of EtOH administration model may affect withdrawal behavior**

We have previously shown that alcohol withdrawal increases negative affective states during protracted withdrawal in mice, some of which can be reversed by MAGL inhibition (Centanni et al., 2019). However, we did not detect clear changes in anxiety-like behaviors at the 72h time point in any assay we conducted, suggesting that the increase in mechanical sensitivity was not secondary to increases in anxiety-like states of mice. In contrast to our findings, mice treated with alcohol five consecutive days a week for 3 weeks via oral gavage exhibited both anxiety-like behavior and mechanical allodynia at 24 hours of alcohol withdrawal (Alongkronrusmee et al., 2016, Xiao et al., 2018). However, this could be partially due to increased stress response seen with oral gavage methods (Brown et al., 2000, Gonzales et al., 2014). Others have reported the emergence of anxiety-like behaviors at different withdrawal time points, such as 5-50 hours into withdrawal, many using alcohol vapor exposure (Baldwin et al., 1991, Rassnick et al., 1993, Metten et al., 2018, Bhattacharya et al., 1995, File, 1994, File et al., 1993, File et al., 1991, Kliethermes, 2005, Lee et al., 2018, Kliethermes et al., 2004, Glover et al., 2019). The findings are not easily compared between studies using different routes of administration and time of testing in withdrawal. Overall, the data presented here agree with literature showing three days as the optimal withdrawal time to induce hyperalgesia in mice following chronic alcohol drinking in a two-bottle choice model (Quadir et al., 2020, Quadir et al., 2021). Under the current experimental conditions of 72-hour withdrawal from alcohol, the effects on mechanical sensitivity and anxiety-like behaviors appear dissociable.

**Possible role of additional endocannabinoid targets**

It will also be important for future studies to investigate additional eCB pharmacological targets. While the current set of experiments focus on the eCB 2-AG, future work should determine how the eCB anandamide (AEA) may influence pain or hypersensitivity during alcohol withdrawal. Inhibition of fatty acid amide hydrolase (FAAH), the primary AEA degrading enzyme, abolished alcohol withdrawal anxiety seen following a single alcohol IP injection (Cippitelli et al., 2008), and FAAH ^-/-^ mice are less susceptible to handling-induced convulsions following chronic ethanol exposure (Vinod et al., 2008). Furthermore, FAAH inhibition reportedly reduced allodynia when co-administered with a COX-2 inhibitor in models of neuropathy and inflammation (Grim et al., 2014), and FAAH inhibitor URB597 was analgesic in both control and ethanol withdrawal mice when injected into the lateral habenula (Fu et al., 2021). Finally, in addition to MAGL, the ABHD6 enzyme hydrolyzes 2-AG in the brain and is located on postsynaptic neuronal elements (Marrs et al., 2010). The literature on the role of this enzyme in pain processing is limited, but there is some evidence inhibition of ABHD6 is antinociceptive in neuropathic pain models (Wen et al., 2018), and may regulate production of 2-AG derived pro-inflammatory prostaglandins (Tanaka et al., 2017). Future studies examining these approaches would be important.

References

Alongkronrusmee D, Chiang T, van Rijn RM (2016) Involvement of delta opioid receptors in alcohol withdrawal-induced mechanical allodynia in male C57BL/6 mice. Drug Alcohol Depend 167**:**190-198.

Atwood BK, Straiker A, Mackie K (2012) CB₂: therapeutic target-in-waiting. Prog Neuropsychopharmacol Biol Psychiatry 38**:**16-20.

Baldwin HA, Rassnick S, Rivier J, Koob GF, Britton KT (1991) CRF antagonist reverses the "anxiogenic" response to ethanol withdrawal in the rat. Psychopharmacology (Berl) 103**:**227-232.

Bhattacharya SK, Chakrabarti A, Sandler M, Glover V (1995) Rat brain monoamine oxidase A and B inhibitory (tribulin) activity during drug withdrawal anxiety. Neurosci Lett 199**:**103-106.

Brown AP, Dinger N, Levine BS (2000) Stress produced by gavage administration in the rat. Contemp Top Lab Anim Sci 39**:**17-21.

Centanni SW, Morris BD, Luchsinger JR, Bedse G, Fetterly TL, Patel S, Winder DG (2019) Endocannabinoid control of the insular-bed nucleus of the stria terminalis circuit regulates negative affective behavior associated with alcohol abstinence. Neuropsychopharmacology 44**:**526-537.

Cippitelli A, Cannella N, Braconi S, Duranti A, Tontini A, Bilbao A, Defonseca FR, Piomelli D, Ciccocioppo R (2008) Increase of brain endocannabinoid anandamide levels by FAAH inhibition and alcohol abuse behaviours in the rat. Psychopharmacology (Berl) 198**:**449-460.

Curry ZA, Wilkerson JL, Bagdas D, Kyte SL, Patel N, Donvito G, Mustafa MA, Poklis JL, Niphakis MJ, Hsu KL, Cravatt BF, Gewirtz DA, Damaj MI, Lichtman AH (2018) Monoacylglycerol Lipase Inhibitors Reverse Paclitaxel-Induced Nociceptive Behavior and Proinflammatory Markers in a Mouse Model of Chemotherapy-Induced Neuropathy. J Pharmacol Exp Ther 366**:**169-183.

Elphick MR, Egertova M (2001) The neurobiology and evolution of cannabinoid signalling. Philos Trans R Soc Lond B Biol Sci 356**:**381-408.

Fernández-Ruiz J, Romero J, Velasco G, Tolón RM, Ramos JA, Guzmán M (2007) Cannabinoid CB2 receptor: a new target for controlling neural cell survival? Trends Pharmacol Sci 28**:**39-45.

File SE (1994) Chronic exposure to noise modifies the anxiogenic response, but not the hypoactivity, detected on withdrawal from chronic ethanol treatment. Psychopharmacology (Berl) 116**:**369-372.

File SE, Andrews N, al-Farhan M (1993) Anxiogenic responses of rats on withdrawal from chronic ethanol treatment: effects of tianeptine. Alcohol Alcohol 28**:**281-286.

File SE, Zharkovsky A, Gulati K (1991) Effects of baclofen and nitrendipine on ethanol withdrawal responses in the rat. Neuropharmacology 30**:**183-190.

Fu R, Tang Y, Li W, Ren Z, Li D, Zheng J, Zuo W, Chen X, Zuo QK, Tam KL, Zou Y, Bachmann T, Bekker A, Ye JH (2021) Endocannabinoid signaling in the lateral habenula regulates pain and alcohol consumption. Transl Psychiatry 11**:**220.

Ghosh S, Wise LE, Chen Y, Gujjar R, Mahadevan A, Cravatt BF, Lichtman AH (2013) The monoacylglycerol lipase inhibitor JZL184 suppresses inflammatory pain in the mouse carrageenan model. Life Sci 92**:**498-505.

Glover EJ, Starr EM, Chao Y, Jhou TC, Chandler LJ (2019) Inhibition of the rostromedial tegmental nucleus reverses alcohol withdrawal-induced anxiety-like behavior. Neuropsychopharmacology 44**:**1896-1905.

Gonzales C, Zaleska MM, Riddell DR, Atchison KP, Robshaw A, Zhou H, Sukoff Rizzo SJ (2014) Alternative method of oral administration by peanut butter pellet formulation results in target engagement of BACE1 and attenuation of gavage-induced stress responses in mice. Pharmacol Biochem Behav 126**:**28-35.

Grim TW, Ghosh S, Hsu KL, Cravatt BF, Kinsey SG, Lichtman AH (2014) Combined inhibition of FAAH and COX produces enhanced anti-allodynic effects in mouse neuropathic and inflammatory pain models. Pharmacol Biochem Behav 124**:**405-411.

Ignatowska-Jankowska BM, Baillie GL, Kinsey S, Crowe M, Ghosh S, Owens RA, Damaj IM, Poklis J, Wiley JL, Zanda M, Zanato C, Greig IR, Lichtman AH, Ross RA (2015) A Cannabinoid CB1 Receptor-Positive Allosteric Modulator Reduces Neuropathic Pain in the Mouse with No Psychoactive Effects. Neuropsychopharmacology 40**:**2948-2959.

Jiang SK, Zhang M, Tian ZL, Wang M, Zhao R, Wang LL, Li SS, Liu M, Li JY, Zhang MZ, Guan DW (2015) The monoacylglycerol lipase inhibitor JZL184 decreases inflammatory response in skeletal muscle contusion in rats. Eur J Pharmacol 761**:**1-10.

Kliethermes CL (2005) Anxiety-like behaviors following chronic ethanol exposure. Neurosci Biobehav Rev 28**:**837-850.

Kliethermes CL, Cronise K, Crabbe JC (2004) Anxiety-like behavior in mice in two apparatuses during withdrawal from chronic ethanol vapor inhalation. Alcohol Clin Exp Res 28**:**1012-1019.

Lee KM, Coelho MA, Class MA, Sern KR, Bocz MD, Szumlinski KK (2018) mGlu5 Receptor Blockade Within the Nucleus Accumbens Shell Reduces Behavioral Indices of Alcohol Withdrawal-Induced Anxiety in Mice. Front Pharmacol 9**:**1306.

Marrs WR, Blankman JL, Horne EA, Thomazeau A, Lin YH, Coy J, Bodor AL, Muccioli GG, Hu SS, Woodruff G, Fung S, Lafourcade M, Alexander JP, Long JZ, Li W, Xu C, Moller T, Mackie K, Manzoni OJ, Cravatt BF, Stella N (2010) The serine hydrolase ABHD6 controls the accumulation and efficacy of 2-AG at cannabinoid receptors. Nat Neurosci 13**:**951-957.

Metten P, Schlumbohm JP, Huang LC, Greenberg GD, Hack WR, Spence SE, Crabbe JC (2018) An alcohol withdrawal test battery measuring multiple behavioral symptoms in mice. Alcohol 68**:**19-35.

Parolaro D (1999) Presence and functional regulation of cannabinoid receptors in immune cells. Life Sci 65**:**637-644.

Patel S, Hill MN, Cheer JF, Wotjak CT, Holmes A (2017) The endocannabinoid system as a target for novel anxiolytic drugs. Neurosci Biobehav Rev 76**:**56-66.

Quadir SG, Rohl CD, Zeabi A, Moore CF, Cottone P, Sabino V (2020) Effect of different standard rodent diets on ethanol intake and associated allodynia in male mice. Alcohol 87**:**17-23.

Quadir SG, Tanino SM, Sami YN, Minnig MA, Iyer MR, Rice KC, Cottone P, Sabino V (2021) Antagonism of Sigma-1 receptor blocks heavy alcohol drinking and associated hyperalgesia in male mice. Alcohol Clin Exp Res 45**:**1398-1407.

Rassnick S, Heinrichs SC, Britton KT, Koob GF (1993) Microinjection of a corticotropin-releasing factor antagonist into the central nucleus of the amygdala reverses anxiogenic-like effects of ethanol withdrawal. Brain Res 605**:**25-32.

Tanaka M, Moran S, Wen J, Affram K, Chen T, Symes AJ, Zhang Y (2017) WWL70 attenuates PGE. J Neuroinflammation 14**:**7.

Thomas A, Okine BN, Finn DP, Masocha W (2020) Peripheral deficiency and antiallodynic effects of 2-arachidonoyl glycerol in a mouse model of paclitaxel-induced neuropathic pain. Biomed Pharmacother 129**:**110456.

Vinod KY, Sanguino E, Yalamanchili R, Manzanares J, Hungund BL (2008) Manipulation of fatty acid amide hydrolase functional activity alters sensitivity and dependence to ethanol. J Neurochem 104**:**233-243.

Wen J, Jones M, Tanaka M, Selvaraj P, Symes AJ, Cox B, Zhang Y (2018) WWL70 protects against chronic constriction injury-induced neuropathic pain in mice by cannabinoid receptor-independent mechanisms. J Neuroinflammation 15**:**9.

Xiao HW, Ge C, Feng GX, Li Y, Luo D, Dong JL, Li H, Wang H, Cui M, Fan SJ (2018) Gut microbiota modulates alcohol withdrawal-induced anxiety in mice. Toxicol Lett 287**:**23-30.
